# Long-term evaluation of combined prosthetic-surgical approach and soft tissue augmentation in the esthetic zone

**DOI:** 10.34172/joddd.2023.40593

**Published:** 2023-11-11

**Authors:** David Nazarian, Viktoriia Olegovna Dzhuganova, Anastasia Nefedkina, Georgy Zakharov, Aleksander Fedosov, Grigoriy Kyalov, Arbak Khachatryan

**Affiliations:** ^1^Maxillofacial and Reconstructive Surgery Department, Federal State Budgetary Institution The National Medical Research Center for Otorhinolaryngology of the Federal Medico-Biological Agency of Russia, Moscow, Russia; ^2^IM Sechenov First Moscow State Medical University, Moscow, Russia

**Keywords:** Esthetic zone, Immediate implant placement, Immediate prosthetic loading, One-stage implant placement, Soft tissue grafting

## Abstract

**Background.:**

There is no standard protocol for immediate implant placement and subsequent loading in the smile zone. We aimed to evaluate the long-term outcomes of simultaneous implant placement, soft tissue grafting, and immediate prosthetic loading in the esthetic zone.

**Methods.:**

Thirty-five implants were placed in the maxillary aesthetic zone. Twenty-two patients were evaluated using the Pink Esthetic Score (PES) and White Esthetic Score (WES). Also, the degree of peri-implant bone resorption and patient survey were applied for the esthetic and functional outcomes.

**Results.:**

The esthetic and harmonizing outcomes were achieved according to the mean total PES/WES value (17.9±2.0). The mean overall PES was 8.5±1.66. The papilla level had the highest mean score (1.8±0.36). Furthermore, the combination of root convexity/color and soft tissue color and texture was one of the key values in evaluating the effectiveness of this method (the mean value was 1.5±0.5). The mesial and distal papillae were 1.6±0.5 and 1.8±0.4, respectively. None of the 35 implants reached below 6 points (which is considered an esthetically unsatisfactory result). The mean WES score was 9.5±0.57. The average degree of total peri-implant bone resorption was 1.05±0.3 mm after 12 months. According to the questionnaire, all the patients smiled without hesitation and were satisfied with the treatment (100%).

**Conclusion.:**

This study showed that restoring one or more teeth in the smile zone using the concept of one-stage implant placement, soft tissue flap augmentation, and loading with provisional crowns was an esthetically successful and predictable method.

## Introduction

 The essential element of implant dentistry is masticatory function and the health and appearance of gums and crowns. Smile design immediately after implantation in the esthetic zone influences patients’ self-confidence. Moreover, a combined approach allows for a reduction in treatment time.^1,2^

 For optimal esthetic and functional dentistry rehabilitation of a patient, the following parameters are considered indispensable: sufficient bone volume, rational implant position, stable and healthy soft tissue around the implant, aesthetic contours of the soft tissue, and an ideal eruption profile.^3,4^

 Peri-implant soft tissue augmentation is important to create attached keratinized mucosa and soft tissue height between implants and achieve esthetic outcomes. Patients with a thin gingival biotype are susceptible to postoperative changes, gingival recession, or the formation of “black triangles.”^5,6^

 In addition, simultaneous implant placement, plastic reconstruction, and placement of crowns immediately after surgery help shorten the duration of prosthetic restoration in contrast to the classic two-stage technique.

## Methods

 We included 22 patients with single or two implants placed in the smile zone. They underwent a combined approach with one-step soft tissue augmentation and provisional crown loading. The total implant number was 35. The group included 10 women and 12 men, aged 20‒70.

 Assessments were performed after 1‒5 years. The implant systems were Nobel Biocare AB (Gothenburg, Sweden) and Renova (Altracor, USA), with a diameter of 3.75‒4.3 mm and a length of 11.5‒13 mm. The provisional crowns were placed immediately after the surgical phase using the transocclusal fixation method. After 4‒6 months, the provisional crowns were replaced with final crowns.

###  Inclusion criteria

 4 mm of bone was present in the apical part of the socket, with stable alveolar ridge walls. The torque during implant placement was 30‒45 Ncm. Patients had no systemic disease that could affect the outcome of the implant or peri-implant plastic surgery (osteoporosis, bisphosphonate medication, coagulation disorders), with treated chronic periodontitis, proper periodontal care, and good oral hygiene.

 The visual-esthetic evaluation was performed using medical perioperative photographs based on Pink and White Esthetic Scores (PES & WES) “before” and “after” the combined treatment (after 12 months), as well as the subjective evaluation of the results using a questionnaire. To analyze the dynamics of the degree of bone resorption, the distance between the implant shoulder and the first contact of the bone with the implant was determined based on orthopantomography (OPG) “before” and “after” (after 12 months) using the Planmeca program (image parameters at a ratio of 1:1, magnification: 150%)

 The data were statistically processed in MS Excel 2019: Quantitative variables were presented as means and standard deviations. Qualitative indicators were presented as absolute and relative values (calculated as percentages).

 The protocol conformed to the Declaration of Helsinki. Each patient received a detailed description of the treatment and gave informed consent.

###  Preoperative analysis

 A conventional dental photo protocol, OPG, and a cone-beam computed tomography system were applied before the surgical treatment. The gingival biotype was also assessed—the presence of a thin biotype was an indicator of soft tissue plastic surgery.^7^ The results were evaluated immediately after implant placement and after one year.

###  Pink Esthetic Score (PES)

 Esthetic and harmonizing soft tissue adaptation was achieved by a comparative analysis with symmetrically standing teeth based on PES, first described by Fürhauser et al.^8^ The evaluation was performed one year after implant placement. The modified PES was based on five variables: mesial papilla, distal papilla, gingival curvature, mucosal level, and root bulge/soft tissue color/texture. Each variable was scored on a scale of 2-1-0, with 2 being the best and 0 being the worst. Medial and distal papillae were scored for completeness, incompleteness, or absence. All other variables were evaluated by comparison with the contralateral reference tooth.

###  White Esthetics Score (WES)

 The WES focuses on the visible part of the implant restoration (i.e., the part of the implant crown that protrudes from the mucosa around the implant). It is based on the following five parameters: overall tooth shape, crown outline and volume, color/shade, surface texture, and transparency.^2,9,10^ All five parameters are assigned a score of 2, 1, or 0, which are evaluated by direct comparison with a natural contralateral reference tooth, the degree of match, or possible mismatch.

###  Patient survey 

 The questionnaire consisted of 5 questions about specific esthetic and functional parameters (Table 1). Subjective evaluation of treatment outcome was assessed one year after treatment. The questionnaire was evaluated according to the recommendations for the test method for measuring subjective or behavioral phenomena.

**Table 1:** Questionnaire

**Question**	**Scoring**
I. Are you ashamed of your smile (smile esthetic satisfaction)?	1. No
	2. Sometimes
	3. Yes, I hide my smile
II. Have you changed diet (using softer texture, difficulties with rough food)?	1. No, the diet remains the same
	2. Yes, I can't eat rough food.
III. Do you have incidents of food stuck between crowns and teeth?	1. Yes
	2. No
IV. Do you routinely use additional oral care products (oral irrigators, floss)?	1. Yes
	2. No
V. Do you have incidents of bleeding gum during brushing teeth in the implant placement area?	1. Yes
	2. No

###  Surgical protocol 

**Figure 1:**
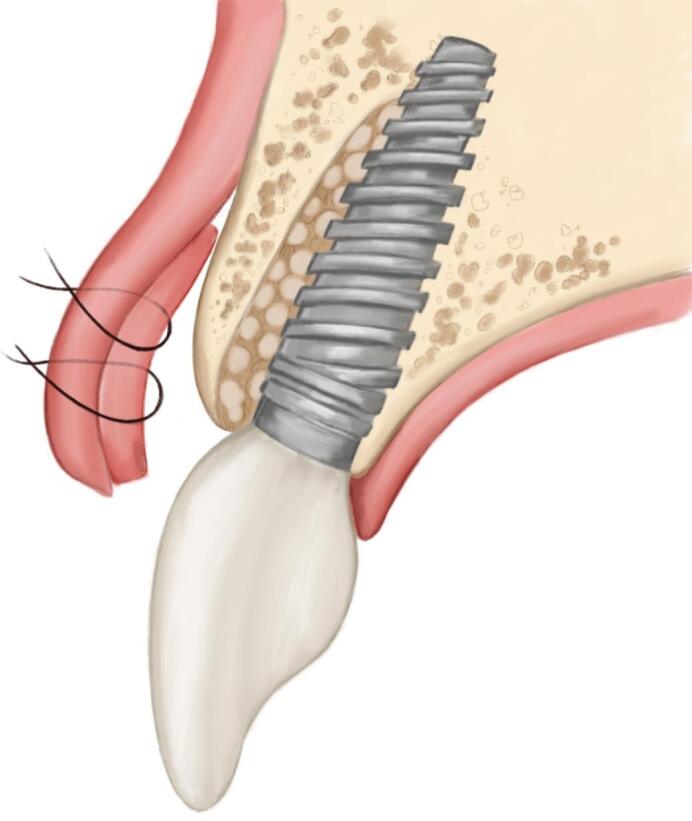
Technique scheme

**Figure 2:**
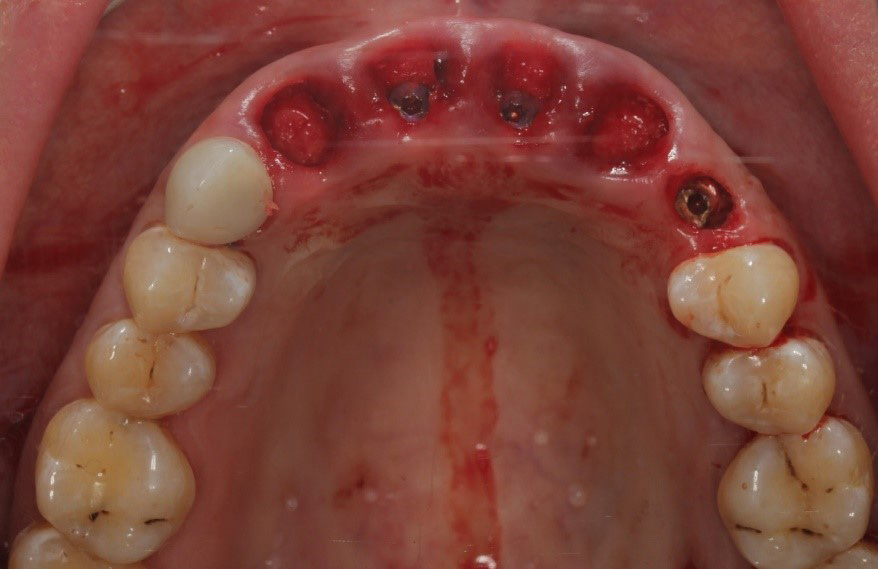
Initial status

**Figure 3:**
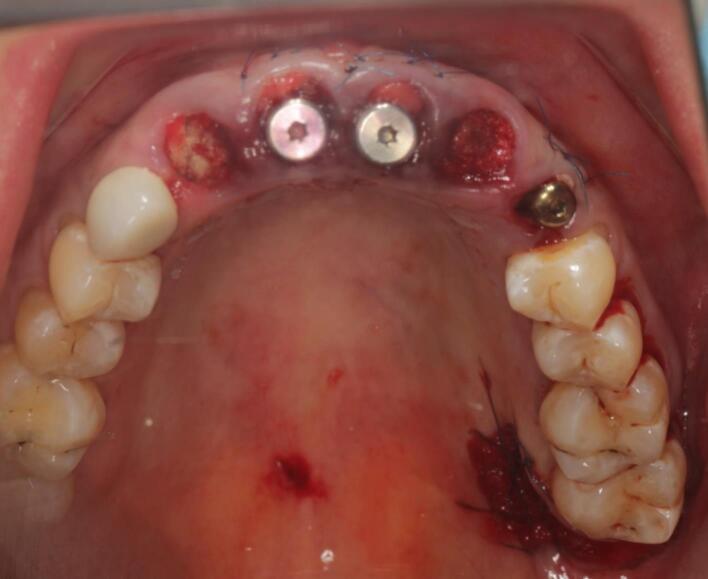
Final status

**Figure 4:**
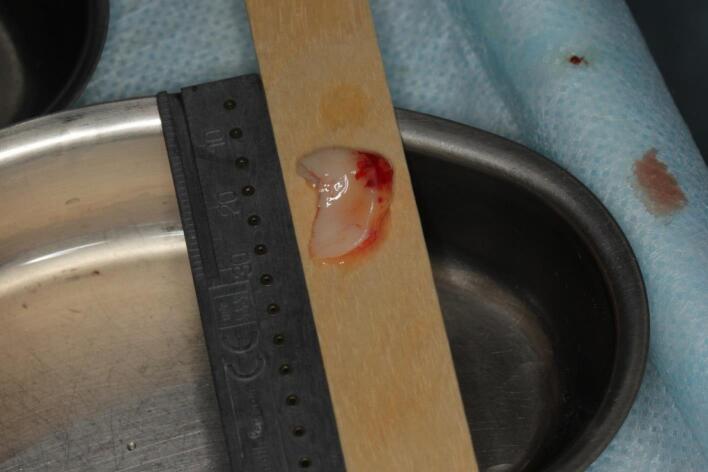
Implant placement

 The procedure included three steps: tooth extraction, immediate implant placement, and peri-implant soft tissue surgery with a connective tissue graft from the maxillary tuberosity (Figures 1, 2, 3, and 4). A pocket was formed on the vestibular aspect of the implant to be placed, and its expansion was limited to preserve the integrity of the hard and soft tissues. Implants were placed according to standard surgical protocol with immediate insertion 3 mm apical to the mid-vestibular mucosal plane with a torque of 30, 45, and 55 Ncm. The bone quality of each patient was assessed during the procedure. Primary stability was achieved by virtue of the palatal wall. In the projection of the maxillary tuberosity, a full-thickness 2 × 2‒3-cm connective tissue graft was harvested and de-epithelialized. The soft tissue graft was fixed in the vestibule with interrupted sutures.

###  Postoperative recommendations

 Systemic antibiotics (amoxicillin + clavulanic acid, two times, 1000 mg/d) were administered peri- (30 minutes before) and postoperatively for 5 days. Postoperative care consisted of rinsing with an 0.2% chlorhexidine bigluconate solution twice daily (60 seconds each) for two weeks without brushing the teeth in the surgical area. Sutures were removed 10‒14 days postoperatively. Weekly checkups were scheduled to monitor oral hygiene and wound healing until sutures were removed.

###  Orthopedic protocol (provisional phase)

**Figure 5:**
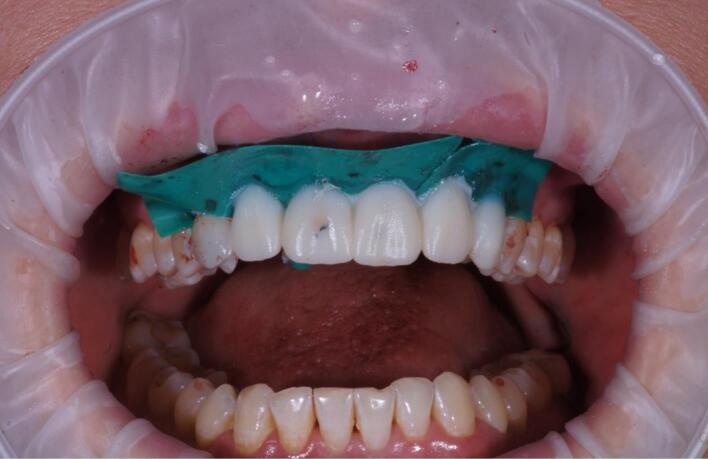
Soft tissue and gingiva former placement

**Figure 6:**
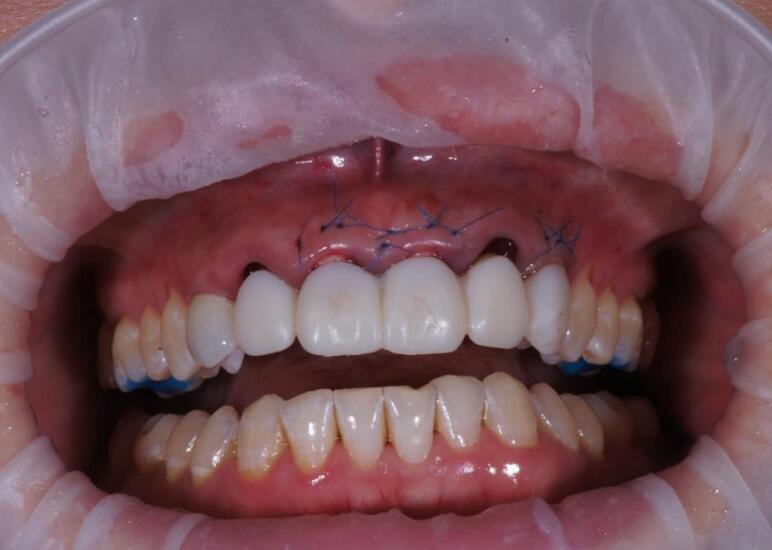
Soft tissue graft

 The provisional crowns were placed immediately after the completion of the entire surgical phase (Figures 5 and 6). A temporary abutment and bis-acrylic material for provisional restorations in combination with a light-curing composite filling material were used for their fabrication. The sterile titanium provisional abutment was isolated with a collagen sponge to retain the blood clot. The alveolus was isolated with a rubber dam to apply the filling material and prevent infection. The structure was completed outside the oral cavity - modernizing the shape of the sub- and supragingival parts of the orthopedic structure to create a proper fit along the upper contour of the socket and create a space for a blood clot to protect the bone structure. All provisional orthopedic prostheses loaded immediately after implantation had a transocclusive fixation method.

###  Final restoration

**Figure 7:**
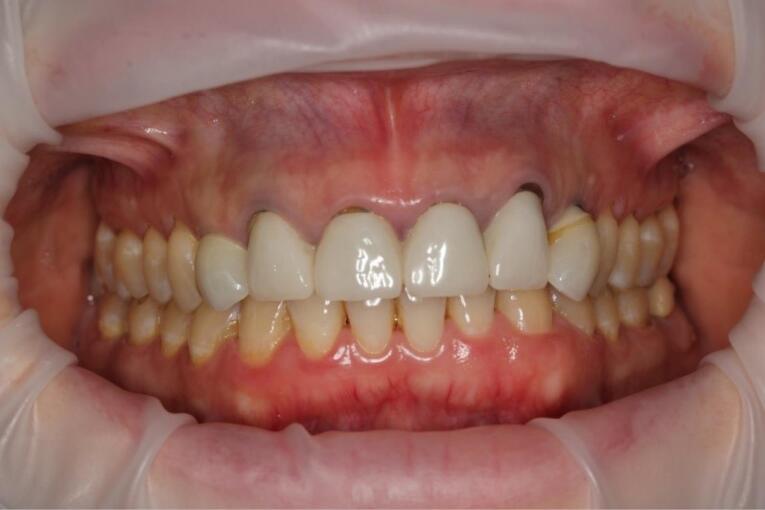
Orthopedic step

**Figure 8:**
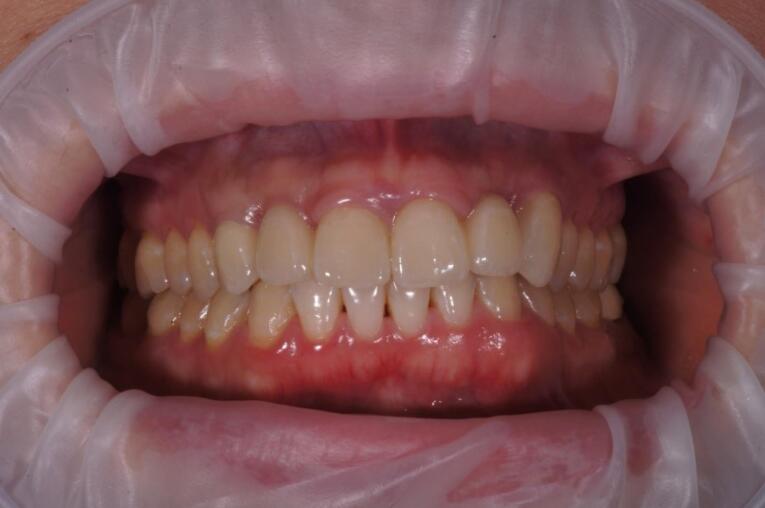
Temporary crowns

**Figure 9:**
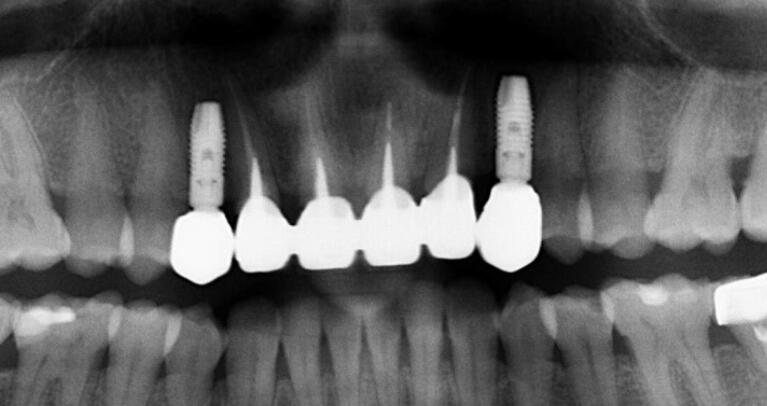
initial panoramic image

**Figure 10:**
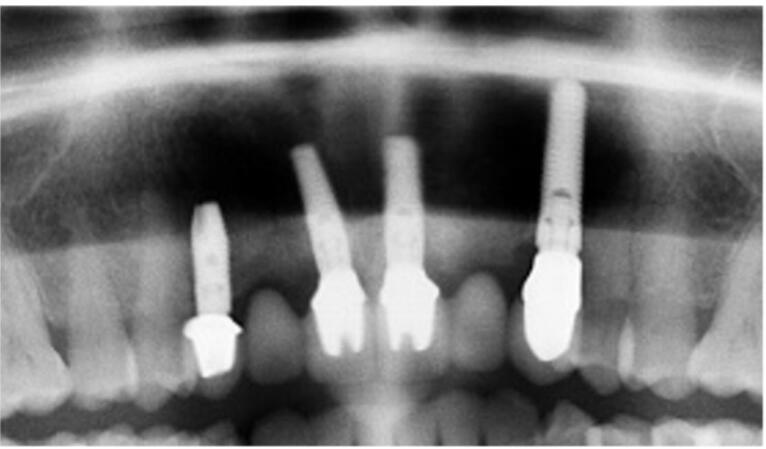
final panoramic image

 After 4‒6 months, the osseointegration and maturation of the gingival flap were completely developed. The soft tissue contour around the dental implant was corrected by fabricating the old provisional crown or making a new crown, considering the desired result. A digital protocol was routinely used to fabricate provisional transocclusal crowns based on dental implants. The provisional crown was used to facilitate processing and control the pressure force. The criterion for proper pressure on the mucosa during profiling was the presence of mild ischemia, which resolved after 10‒15 minutes. If ischemia persisted, the volume of the provisional crown was corrected to achieve the desired effect. After the final shape of the gingival profile was formed around the implant, a final prosthesis was fabricated. Both digital and analog techniques were used to fabricate final crowns. The impression copings or scan markers were individualized by copying the soft tissue contour onto them immediately after removing the provisional crown with a liquid composite. In the classic variant of fabricating provisional structures, a combination of an individualized zirconia abutment and a ceramic crown was used. The type of ceramic crown was selected depending on each patient’s occlusal and esthetic characteristics (Figures 7, 8, 9, and 10).

## Results

 In this study, 35 implants were placed in an esthetic zone. Twenty-three implants were positioned in the projection of teeth #11 and #21. None of the patients experienced complications related to osseointegration (implant rejection) or infection.

###  Bone resorption after 12 months

 A slight bone resorption was detected in the distal papillae. The average distance between the implant shoulder and the first contact of the bone with the implant was 1.05 ± 0.3 mm after 12 months. In the projection of the mesial papilla, the parameter was 0.96 ± 0.5 mm.

###  Esthetic evaluation of treatment 

 The mean total PES/WES value was 17.9 ± 2.0. The average total value of PES was 8.5 ± 1.66. The parameter PES – papilla level (1.8 ± 0.36) had the highest mean scores, while the combination of variable root convexity/color and soft tissue color and texture (1.5 ± 0.5) was one of the key values in evaluating the effectiveness of this method; 18 of 35 implant sites achieved a maximum score of 2. The mesial papilla was 1.6 ± 0.5 and the distal papilla was 1.8 ± 0.4. None of the 35 individual implants achieved < 6 points (esthetically an unsatisfactory result). The mean WES score was 9.5 ± 0.57. It should be noted that esthetic scores of crown surface texture and translucency achieved maximal values in all the patients. The mean overall score of tooth form evaluation was 1.9 ± 0.3, and crown color with a natural contralateral reference tooth was 1.83 ± 0.37 (Table 2).

**Table 2:** Detailed PES and WES of all 35 included implants

**Patient**	**Implant site**	**PES**						**WES**						**PES+WES**
		**Mesial papilla**	**Distal papilla**	**Curvature of facial mucosa**	**Level of facial mucosa**	**Root convexity, soft tissue color and texture**	**Total PES**	**Tooth form**	**Tooth volume/ outline**	**Color (hue/value)**	**Surface texture**	**Translucency and characterization**	**Total WES**	**Total PES+WES**
1	11	1	2	2	1	1	7	2	1	2	2	2	9	16
	21	1	2	2	1	1	7	2	1	2	2	2	9	16
2	21	1	2	1	2	2	8	2	2	2	2	2	10	18
3	21	2	1	2	2	2	9	2	2	2	2	2	10	19
4	21	2	2	2	2	2	10	2	2	2	2	2	10	20
	11	2	2	2	2	2	10	2	2	2	2	2	10	20
5	12	2	2	2	2	2	10	2	2	2	2	2	10	20
	13	2	2	2	2	2	10	2	2	2	2	2	10	20
6	11	2	1	2	2	1	8	2	2	2	2	2	10	18
	12	1	2	2	2	1	8	1	2	2	2	2	9	17
7	11	2	2	2	2	2	10	2	2	2	2	2	10	20
	21	2	1	1	1	1	5	1	1	1	1	1	5	10
	23	2	1	1	1	1	5	1	1	1	1	1	5	10
8	21	1	1	1	1	1	5	1	1	1	1	1	5	10
9	22	2	2	2	1	1	8	2	2	1	2	1	8	16
10	22	1	1	2	1	0	5	2	2	1	1	0	6	11
11	21	2	2	1	2	1	8	1	1	1	1	0	4	12
12	11	1	1	1	1	1	5	1	1	1	1	1	5	10
	21	1	1	1	1	1	5	1	1	1	1	1	5	10
13	11	2	2	2	2	0	8	2	2	2	2	2	10	18
	22	2	2	2	2	0	8	2	2	2	2	2	10	18
14	11	2	2	1	1	0	6	0	0	0	0	0	0	6
	12	2	2	1	1	0	6	0	0	0	0	0	0	6
15	22	1	1	1	1	1	5	1	1	1	1	1	5	10
	13	1	1	1	1	1	5	1	1	1	1	1	5	10
16	11	1	1	1	1	1	5	1	1	1	1	1	5	10
17	11	1	1	1	1	1	5	1	2	1	1	1	6	11
	21	1	1	1	1	1	5	1	2	1	1	1	6	11
18	11	1	2	2	1	1	7	1	1	1	1	1	5	12
	21	1	2	2	1	1	7	1	1	1	1	1	5	12
19	21	1	1	1	1	1	5	2	2	2	2	2	10	15
20	22	2	1	2	1	2	8	2	2	2	2	2	10	18
21	21	2	2	2	2	2	10	2	2	2	2	2	10	20
	22	2	2	2	2	2	10	2	2	2	2	2	10	20
22	21	2	2	2	2	2	10	2	2	2	2	2	10	20

PES: Pink Esthetic Score, WES: White Esthetic Score.

###  Questionnaire data 

 All the patients smiled without any hesitation and were satisfied with the treatment. The patients responded positively to not changing their diet without incidents of food impaction between crowns and teeth; 21% of patients routinely used additional oral care products (oral irrigators, dental floss). Only one person complained of bleeding gums during toothbrushing (4.5%).

## Discussion

 During dental implant placement in the smile area, the esthetic outcomes are as important as the restoration of the chewing function.^1,9,11,12^

 Immediate dental implant placement is considered a “gold standard” when esthetics is a priority. There are few contraindications of immediate dental implants, including severe bone atrophy and the presence of severe inflammation.^1,7,13,14^ One-step approach shortens the treatment duration compared to the classic two-stage technique. Using immediate loading with provisional crows, this technique allows to achieve good preliminary results without compromising esthetics. Provisional crowns make it possible to create a protective barrier for the implant and the gingival graft, simultaneously maintaining the contour of the extracted tooth gingival tissues.^15^ The prosthetic crown supports the soft tissue graft and promotes its ingrowth, protecting the transplant from injury. Moreover, orthopedic construction allows the formation of soft tissue contours.^4,5,16^

 There are several areas for harvesting soft tissue grafts, from hard palate to maxillary tuberosity, which is considered a reliable and effective technique for augmenting soft tissue defects around the implant.^17,18^

 We must mention that optimal results were achieved in all cases, with PES (mean score = 8.5) and WES (mean score = 9.5). None of the 34 implants scored < 6, confirming a good esthetic prognosis of the surgical and orthopedic protocols.

 Achieving good results is related to the influence of local anatomical conditions, the applied combined surgical technique, and the regeneration of bone and soft tissue defects around the implants, which are usually present at implant placement sites after removal.^19^

 The main goal of the applied surgical protocol is to predictably contour the soft tissue to obtain esthetic results, especially the prevention of mucosal recession.

 Two PES parameters, facial mucosal curvature and height, were evaluated high (mean = 1.7 and 1.8, respectively), indicating that this objective was met with a good prognosis. The highest possible combined PES /WES score of 20 was achieved in seven patients (32%), indicating an identical match between the peri-implant soft tissue condition and the clinical crown of a single implant with the corresponding characteristics of the contralateral natural tooth.

 Bone destruction at 12 months is not very pronounced (the average bone resorption rate is 0.2 ± 0.4 distally and 0.14 ± 0.4 mesially), which correlates with the good results obtained in the evaluation of the esthetic aspects of the soft tissue around the implants and the provisional crowns, as well as the subjective assessment of the results of the procedures by the patients.^20^

## Conclusion

 This study has shown that restoring one or more teeth in the smile zone using one-stage implant placement, soft tissue flap augmentation, and loading with provisional crowns is an esthetically successful and predictable method. This technique makes it possible to preserve and improve the esthetic and functional outcome.

## Acknowledgments

 This review article received no specific grant from any funding agency in the public, commercial, or not-for-profit sectors.

## Competing Interests

 We declare no conflicts of interest involved in this study.

## Ethical Approval

 This study was approved by the Medical Ethics Committee of Hokkaido University No. 016-0170.
